# Microvascular obstruction persistence after early-reperfused myocardium infarction by CMR: analysis of first week and six months after STEMI

**DOI:** 10.1186/1532-429X-13-S1-P157

**Published:** 2011-02-02

**Authors:** Amarino C Oliveira, Denise M Moreira, Carlos E Rochitte, Suzana A Silva, Hans F Dohmann

**Affiliations:** 1 Pro Cardiaco Hospital, Rio de Janeiro, Brazil; 2 Heart Institute -InCor- University of Sao Paulo Medical School, Sao Paulo, Brazil; 3 Teaching and Research Center of Pro Cardiaco Hospital/Procep, Rio de Janeiro, Brazil

## Introduction

Detection of microvascular obstruction (MO) with gadolinium first-pass perfusion (FP) and late gadolinium enhancement (LGE) MRI predicts outcomes of STEMI patients. Previous data indicate the presence of MO early after MI correlates to worse prognosis and left ventricular (LV) remodeling. Nonetheless, FP and early hypoenhancement on LGE may persist after reperfused STEMI for a variable period of time.

## Purpose

We investigate whether the persistence of MO by CMR was related to worse LV remodeling at 6 month after STEMI.

## Methods

We retrospectively analyzed 27 patients admitted with successfully reperfused STEMI and with infarct size > 10% of LV mass, who were enrolled in a stem cell trial. Baseline MRI was performed after the successful percutaneous coronary reperfusion, during first week, and repeated three and six months in the follow up. CMR exam included cine-MR using SSFP, FP and LGE acquisitions. Infarct size, LV volumes, mass and ejection fraction were measured by planimetry. Presence of hypoenhanced areas on LGE or perfusion defects on FP images within the infarct area were interpreted as MO. Patients were classified as absent MO (group 1), with MO on first CMR only (transient MO, group 2) and with MO on both studies (persistent MO, group 3). ANOVA was used to compare LV parameters at 6 month within the groups.

## Results

From 27 patients, 6 were in group 1, 8 in group 2, and 13 in group 3. LV ejection fraction at six month was significantly lower in persistent MO group compared to transient and absent MO (43.6±9.7% vs 47.1±7.9% vs 62.5±15.7%, p=0.006 by ANOVA, p<0.05 for group 1 vs 2 or 3 by Bonferroni sub-test). The percent increase in LVEF from baseline to 6 month was also lower for group 3 than for group 2 or 1 (0.9±18.9% vs. 9.5±28.8% vs. 48.4±50.0%, p=0.02). EDV was not statistically higher in group 3 compared to 2 and 1 (156.7±46.5ml vs. 134.7±48.6ml vs. 130.0±26.9ml, p ns). Infarct size was larger in persistent MO group compared to the other 2 groups (32.9±9.5% vs. 27.2±14.3% vs. 18.8±10.2% of LV mass, p<0.05)

## Conclusions

Persistence of microvascular obstruction defined as first-pass perfusion defect or early hypoenhancement core within the infarct was associated to worse left ventricular ejection fraction, remodeling and myocardial infarction. The correlation of MO persistence with prognostic surrogates, such as ventricular function and infarct size, warrants further investigation on the influence of MO persistence on the clinical events and prognosis.

